# Household Food Insecurity and Its Association with Nutritional Status of Children 6–59 Months of Age in East Badawacho District, South Ethiopia

**DOI:** 10.1155/2017/6373595

**Published:** 2017-03-16

**Authors:** Bealu Betebo, Tekle Ejajo, Fissahaye Alemseged, Desalegn Massa

**Affiliations:** ^1^Department of Public Health Emergency Management, Hadiya Zone Health Department, Hosanna, Ethiopia; ^2^Department of Plan Monitoring and Evaluation, Hadiya Zone Health Department, Hosanna, Ethiopia; ^3^Department of Epidemiology, College of Public Health and Medical Sciences, Jimma University, Jimma, Ethiopia

## Abstract

*Background*. Ethiopia has one of the highest child malnutrition rates in the world. Food insecurity is one of the determinant factors of malnutrition in developing countries; however its role remains unclear.* Objective*. To assess household food insecurity and its association with the nutritional status of children 6–59 months of age in East Badawacho District, South Ethiopia.* Methods*. A community based cross-sectional study was conducted from February 20 to 30, 2014 on a sample of 508 mother/child pairs of 6–59-month-old children. Sample households with eligible children were selected using systematic random sampling technique. Both bivariate and multivariate analysis were used to identify factors associated with nutritional status of children. *P* value of <0.05 was considered as statistically significant.* Result*. The prevalence of household food insecurity was 75.8%. The prevalence rates of stunting, underweight, and wasting among children were 45.6%, 26.3%, and 14.6%, respectively. Household food insecurity was significantly associated with underweight (AOR = 3.82; CI = 1.78–8.19) and stunting (AOR = 6.7; CI = 3.71–12.1) but not with wasting.* Conclusion and Recommendation*. Household food insecurity and the prevalence rates of stunting, underweight, and wasting, among children 6 to 59 months, were high. Intervention programs should focus on improving household food insecurity and nutritional status of children.

## 1. Background

Nutrition is a cornerstone that affects and defines the health of all people, rich and poor. Conversely, malnutrition makes us all more vulnerable to disease and premature death [1, 2]. It is a devastating problem, particularly for the poor and unprivileged as poverty is a fundamental cause of household food insecurity and consequently malnutrition which continues to be one of the major and most pressing health problems affecting children and adults [2, 3].

Malnutrition literally means “bad nutrition” and technically includes both over- and undernutrition. In the context of developing countries, undernutrition is generally the main issue of concern, though industrialization and changes in eating habits have increased the prevalence of overnutrition. Within the context of World Food Program (WFP), malnutrition refers to undernutrition unless otherwise specified [4].

Hence, malnutrition is usually the result of a combination of inadequate dietary intake and infection [5]. Children are most at risk, because they are more vulnerable to adverse environments and respond rapidly to dietary changes; they are also more at risk of becoming ill which will result in weight loss. Consequently, their nutritional status is considered a good gauge for population-based malnutrition. Therefore, the survey results of the under-5-years population are used to draw conclusions about the situation of the whole population, not just of that age group [4].

Undernutrition in children can manifest itself in several ways, and it is most commonly assessed through the measurement of weight and height [5].

Stunting, the anthropometric index height for age, reflects linear growth achieved pre- and postnatally with its deficits indicating long-term, cumulative effects of inadequate nutrition and/or health. Wasting describes a recent and severe process that has produced a substantial weight loss, usually as a consequence of acute shortage of food and/or severe disease. The anthropometric index weight for height reflects body weight relative to height. Underweight, the anthropometric index weight for age, represents body mass relative to age. It is influenced by the height and weight of a child and is thus a composite of stunting and wasting [6].

Multiple and interrelated determinants are involved in why malnutrition develops [7], And food insecurity is one among the key risk factors. By a widely accepted definition, a household is considered food insecure if it has limited or uncertain physical and economic access to secure sufficient quantities of nutritionally adequate and safe foods in socially acceptable ways to allow household members to sustain active and healthy living [8, 9].

Worldwide, over 10 million children under the age of 5 years die every year from preventable and treatable illnesses despite effective health interventions. More than one-third of these deaths are caused by malnutrition [10]. MDG-1 is targeted at reducing undernutrition by half. However, in the developing countries 146 million children under 5 suffer from undernutrition which is one of the main factors that causes malnutrition [11].

In Ethiopia, one of the world's poorest countries with low levels of development, many people live in conditions of chronic hunger with a low average daily energy supply [12]. The country also faces serious and growing food insecurity problem, affecting as much as 45% of the population [13]. Malnutrition in children is one of the most serious public health problems in Ethiopia and the highest in the world [14]. The country has the second highest rate of malnutrition in Sub-Saharan Africa [15] and with high under-five mortality rate (88/1000 live births) which is mainly due to infection and malnutrition [16].

According to 2011 Ethiopian DHS 44.4%, 9.7%, and 28.7% of children under five years of age are stunted, wasted, and underweight, respectively. Similarly, In SNNPR (Southern Nation Nationalities and Peoples Region) prevalence of child malnutrition indicates that 44.1, 7.6%, and 28.3% of children are stunted, wasted, and underweight, respectively [16].

HHFI and childhood malnutrition is highly prevalent in SNNPR, particularly in the study area; however there is no a single study conducted on the association between food security and nutritional status of children. Accordingly, this study explored the prevalence, as well as the association between, household food insecurity and nutritional status of children 6–59 months of age in East Badawacho District, Hadiya Zone, Southern Ethiopia.

## 2. Methods and Materials

### 2.1. Study Area and Period

Community based cross-sectional study design was employed using quantitative approach in East Badawacho District, from February 20 to 30, 2014. The district is one of ten administrative districts found in Hadiya Zone of Southern Ethiopia. The district has thirty-nine rural kebeles (smallest administrative units). Its annual rain fall amount ranges from 800 mm to 1300 mm. The majority of the population economy depends on traditional agriculture; the main crop produced in the area is maize.

### 2.2. Sample Size Determination and Sampling Procedure

#### 2.2.1. Sample Size Determination

To determine the children to be included in the study different proportions were identified in order to get a larger sample size.

Based on the 2011 EDHS report the prevalence of stunting, underweight, and wasting in SNNPR is 44.1%, 28.3%, and 7.6%, respectively [16]. And in the study conducted in 2012 in Wolayita, SNNPR showed that 74.2% households face food insecurity [17]. And also in another similar study which is conducted in iie-ife, Nigeria revealed that the proportion of underweight in food secure and insecure household was 8.3% and 17.2%, respectively [18].

So the sample size was determined using Epi Info version 7 based on the following assumption.

*Expected Prevalence*. The prevalence which gives larger sample size was taken, that is, proportion of underweight in food insecure household *p*_1_ = (17.2%) and proportion of underweight in food secure households *p*_2_ = (8.3%) iie-ife, Nigeria.

*Confidence interval (CI)* = 95%, which means *α* is set at 0.05 and *Z*_*α*/2_ = 1.96 (value of *Z* at *α* 0.05 or critical value for normal distribution at 95% CI). And* power* is 80%.

As the main aim of the study is to compare the nutritional statuses of children between food secure and food insecure households, a two-population-proportions formula was used to determine the sample size.

Then the number of children that are included in the study was 484 (242*∗*2) and by adding a 5% of the sample for nonresponse rate, the final sample size was 508 children/caregiver pairs.

#### 2.2.2. Sampling Technique/Procedure

There are 39 kebeles (smallest administrative units) in East Badawacho District. To generate a sampling frame for each kebele, households with children 6–59 months of age were identified in each kebele, from Health Post family folder. The calculated sample (508) was proportionally allocated to all kebeles. Sample households with eligible children were selected from the sampling frame of each kebele using simple random sampling technique. When there was more than one child present in the selected household, only the youngest child was selected.

### 2.3. Data Collection Instrument and Procedure

#### 2.3.1. Data Collection Instrument

Structured and uniform questionnaire was administered to the caregiver of the study children.* Household Food Insecurity Access Scale (HFIAS)* Measurement Tool which consists of 9 items developed by the Food and Nutrition Technical Assistance (FANTA) project was used to assess the household food insecurity status of households.

Anthropometric measurement on weight and height was taken from children aged 6–59 months. Height of the child was measured using measuring board. Their weight was measured using Salter spring scale.

#### 2.3.2. Data Collection Procedure

Ten data collectors with diploma in clinical nursing and 3 supervisors with degree in clinical nursing or health officer who had been involved in other similar field surveys were recruited.

A face to face interview was made with the caregivers of the child using a pretested structured questionnaire.

*Anthropometry*. Height of infants aged 6–23 months was measured in a recumbent position. Height of children 24 months and older was measured in a standing-up position. Weight of the lightly clothed infants and children was measured to the nearest 10 g.

Data validity and reliability was maintained through close supervision of enumerators by the principal investigator.

### 2.4. Data Processing and Analysis

The collected data was entered into Epi-Data 3.1 and exported into SPSS version-16 statistical software for analysis. WHO Anthro version 3.0.1, 2007, software was used to convert the anthropometric data into *Z*-scores of the indices, HAZ, WHZ, and WAZ.

Bivariate analysis (binary logistic regression) was carried out to select variables for multivariable model. Multivariable logistic regression analysis was carried out to identify the most important predictors of nutritional status of children 6–59 months of age controlling the effects of confounding variables. And *P* value of 0.05 was taken as cutoff point to label the significance of the variables. The strength of association was measured by 95% confidence interval (crude/adjusted odd ratio). Those variables with *P* value ≤ 0.2 in the bivariate analysis were inputs for multivariable logistic regression analysis and multicolinearity between independent variables was checked, and then final model was constructed using enter logistic regression method. All models are checked for their fitness using Hosmer and Lemeshow goodness of fit test.

### 2.5. Data Quality Management

To ensure the quality of data, the questionnaire was developed in English and is interviewer-administered and was translated to the local languages (Amharic and Hadiyisa); then their consistency was checked by another person who speaks both languages. The questionnaire was back-translated to English to check for its conceptual equivalence. And variables incorporated in the questionnaire were adapted from different instruments that were used for the assessment of similar studies [16, 18–22].

A two-day intensive training was given to the selected data collectors by the principal investigator. The training covered study objectives, a thorough review of the questionnaire, the use of survey instruments, interview techniques, and directions on how to administer the structured questionnaire and how to take anthropometric measurements and ethics during field work in line with predesigned training module.

Prior to the actual data collection, the questionnaire was pretested using 5% of the sample from a similar population who were not included in the main study. The questionnaire was modified based on the pretest result accordingly.

Measurement was taken using UNICEF's standard instruments of weighing scale and height board and was routinely checked and adjusted to maintain its accuracy. Calibration of the indicator against zero reading was checked following weighing every child.

### 2.6. Ethical Consideration

Letter of ethical clearance was obtained from ethical review committee of Jimma University College of Public Health and Medical Sciences.

Informed consent was obtained from study participant. All the interviews were made with strict privacy after getting informed consent from the respondents by ensuring the confidentiality of the responses. Thus, name and address of the interviewees were not recorded in the questionnaire. They were also informed that they have full right to discontinue or refuse to participate in the study. For this purpose, a one-page consent letter was attached to the cover-page of each questionnaire stating the general purpose of the study and issues of confidentiality which was discussed by data collectors before proceeding with the interview.

## 3. Result

### 3.1. Demographic and Socioeconomic Characteristics of Parents

A total of 508 households, having at least one child aged 6–59 months, were included in the survey. Data for 12 children were incomplete; thus final analysis was based on 496 mother/child pairs with response rate of 97.6%.

The mean age of the mother was 30 years (SD of 4.8) and the majority 355 (71.6%) of the mothers are in the age group of 25–34. Among the respondents, 447 (90.1%) of the households were male headed. The mean family size was 6.7 with SD of 2. Out of the respondents 147 (29.6%) had a family size less than five. Among the households 262 (52.8%) of them had more than 2 under-five-years children.

The distribution on educational status of the respondents indicates that 217 (43.8%) of mothers had no education and 203 (40.9%) completed primary education and majority (411) (82.9%) of them were housewives. Two hundred and forty-four (49.3%) of the fathers completed primary education and 263 (53%) of them were farmers. Regarding family wealth 20% and 20.4% of the households were very poor and poor, respectively (Table 1).

### 3.2. Obstetric Characteristics of the Mother

Regarding obstetric character of mothers, majority of the mothers accounting for 446 (89.9%) are married. And their average age at marriage was 19.6 (SD of 2.6). Average number of children born to a mother was 4.1 (SD of 2) and 198 (39.9%) of mothers gave birth to more than 5 children and 349 (70.4%) of them visited ANC service when they had the index child.

The health status of 410 (82.7%) mothers was good during their pregnancy and 346 (69.8%) of the mothers have not consumed extra food during the pregnancy of the index child (Table 2).

### 3.3. Demographic and Health Related Characteristics of the Child

From the total children included in the survey 245 (49.4%) were male and the mean age of the children was 26.4 months with SD of 13.2 (Table 3).

Place of delivery for 413 (83.3%) of the children is at home and 372 (75.0%) of the deliveries were attended by Traditional Birth Attendants. For 234 (47.2%) of the children their size at birth was average and 85 (17.1%) had small size during birth.

From the total children 331 (66.7%) had diarrhea, 171 (34.5%) had fever, and 66 (13.3%) had other serious illness two weeks prior to the survey and 21 (4.2%) had measles 6 months prior to the survey (Table 3).

### 3.4. Child Caring Practice

As indicated in Table 4, 419 (84.5%) had breastfeed and 218 (44.0%) of them were initiated breastfeeding practice immediately after birth. In addition, 270 (54.4%) of the children were fed colostrum and about 301 (60.7%) of children started complementary feeding at the age of 6–8 months, and 173 (34.9%) of them were feeding more than four times per day; 115 (27.4%) of children continued breastfeeding for more than 24 months. Eighty-one (16.3%) had received prelactation of food or fluids like butter, milk, or water.

Concerning immunization majority 453 (91.3%) of the children had received immunization, 441 (88.9%) were supplemented with vitamin A, and 286 (57.7%) of them were dewormed 6 months preceding the survey (Table 4).

### 3.5. Household Food Insecurity Status

Household food insecurity status was measured using FANTA HFIAS tool which consists of nine occurrence questions that represent a generally increasing level of severity of food insecurity (access) and nine “frequency-of-occurrence” questions that are asked as a follow-up to each occurrence question to determine how often the condition occurred one month preceding the survey.

It is indicated in Table 5 that 64 percent of the respondents reported to have worried about food shortage during the last four weeks; 66.1 percent reported inability to eat the preferred food; 66.5 percent reported to have eaten limited variety of food; 55.9 percent ate food that they really do not want to eat and were unable to eat the preferred variety of food due to lack of adequate resources; 62.3 percent reported that their household members have eaten smaller amount of food; 66.1 percent missed the number of meals per day; 32.3 percent reported that they have no food of any kind to eat; 10.7 percent reported sleeping without eating food; and 5.8 percent reported to have spent the day and night without eating any food.

Nearly 9 percent of the households enrolled in food aid program in the past 1 month preceding the survey.

From the total household included in the survey more than three-fourths of households (376) (75.8%) experienced some degree of food insecurity in the one month preceding the survey. Among these households 62 (12.6%) were mildly food insecure, 162 (32.2%) were moderate, and 152 (31.0%) were severely food insecure (Table 5).

### 3.6. Nutritional Status of Children

The overall malnutrition prevalence among under-five children was 26.3% for underweight, 45.6% for stunting, and 14.6% for wasting. In addition, the proportions of malnutrition by its degree of severity (10.5%, 26.8%, and 8.0%) were severely underweight, stunted, and wasted, respectively, while 15.7%, 18.8%, and 6.6% were moderately underweight, stunted, and wasted, respectively. Regarding O-edematous malnutrition, 11 (2.2%) children had bilateral O-edema (Table 6).

### 3.7. Household Food Insecurity and Nutritional Status of Children

Regarding distribution of nutritional status of children by household food insecurity status, the majority of malnourished children were found in food insecure households, 114 (89.7%), 205 (90.7%), and 54 (76%) for underweight, stunting, and wasting respectively.

The mean HAZ, WAZ, and WHZ were all negative, suggesting a generally poor nutritional status of the children in the study area. The mean HAZ, WHZ, and WAZ were lower for children from food insecure households than for children from food secure households (*P* < 0.05). However, the mean WHZ has no significant difference between the two groups. Similarly the prevalence rates of stunting and underweight were slightly higher among children from food insecure households than among children from food secure households. However, the prevalence of wasting among children did not differ significantly between food insecure (14.8%) and food secure households (14.1%) (Table 7).

As the main aim of the study was to see the independent association of HHFI with nutritional status of children, both bivariate and multivariable logistic regression analysis were performed to see the association between household food insecurity and the three indices of nutritional status of children controlling the effect of other independent variables.

First bivariate analysis was done for all independent variables separately with each outcome variable. *P* value of <0.2 was used as a cutoff point to select variables. Then those variables which are important predictors and showed association (*P* < 0.2) with the nutritional status (underweight, stunting, and wasting) of children were selected and entered into multivariable analysis, separately for each outcome. And *P* value of 0.05 was taken as cutoff point to label the significance of the variables in multivariable analysis. According to this three models were developed for the three outcomes variables.

During bivariate analysis nine variables were found to be associated (*P* < 0.2) with underweight and four of them came out to be risk factors in multivariable analysis (Table 9).

For stunting, seven variables with *P* value < 0.2 were selected in bivariate analysis but only three of them showed independent association in multivariable analysis (Table 10). Similarly eight variables had *P* < 0.2 with wasting in bivariate analysis but multivariable analysis identified five of them to be significantly associated with wasting (Table 11). There was no collinearity between independent variables and Hosmer and Lemeshow goodness of fit test was also performed for all models and yielded significant result (*P* > 0.05).

During both bivariate and multivariable analysis, household food insecurity was associated with stunting and underweight but not with wasting (Table 8). Children living in food insecure households had higher risk to be underweight and stunted than children living in food secure households. Children living in food insecure households were 3.82 times more likely to be underweight compared to children living in food secure households (AOR = 3.82; CI = 1.78, 8.19) and the risk of being stunted, for the children living in food insecure household, was 6.7 times higher than those children living in food secure households (AOR = 6.7; CI = 3.71, 12.1).

Other important predictors were also identified which showed significant association with nutritional status of children (Tables 9, 10, and 11).

### 3.8. Other Factors Associated with Nutritional Status of Children

To see the independent association of HHFI with nutritional status of children it was adjusted with other variables in multivariable analysis, and some of these variables were also found to be the risk factors for nutritional status of children.

From the variables which were entered into multivariate analysis diarrhea in the last 2 weeks, ANC visit of the mother, health status during pregnancy, and HHFI were significantly associated with underweight (Table 9).

The risk of being underweight was 2.5 times higher for children who had experienced diarrhea in the last 2 weeks than in children who had not (AOR = 2.5; CI = 1.52–4.13). The risk of being underweight for children whose mother did not receive ANC service was 2.8 times higher than children whose mothers did (AOR = 2.8; CI = 1.66–4.7) and children whose mothers had no good health status during pregnancy were 2.23 times more likely to be underweight than children whose mothers had good health status during pregnancy (AOR = 2.23; CI = 1.27–3.94).

In addition, extra food during pregnancy and mothers age have shown significant association with underweight in bivariate analysis (*P* < 0.05). The risk of being wasted was 1.66 times higher among mothers of children who had not consumed extra food during pregnancy than those mothers of children who had consumed extra food during pregnancy (COR = 1.66; 95% CI = 1.03–2.26) (Table 9).

From the variables which were identified as candidates and entered into multivariable regression analysis (to identify potential predictors) for stunting, child age, initiation of complementary feeding, and HHFI were found to be significantly associated with stunting.

Stunting was high in the age group of 12–23 months (26.5%) and 36–47 (28.3%); however only children in the age group of 36–47 months showed significant association with stunting. Children in the age group of 36–47 months were about 2 times more likely to be stunted than children aged 6–11 months (AOR = 2.17; 95% CI = 1.11–4.28). It was also observed that children who had initiated complementary feeding early (before 6 month) were 2.41 times more at risk to be stunted than children who had started complementary feeding timely (6–8 months) (AOR = 2.41; CI = 1.36–4.27) (Table 10).

Among the variables which were entered into multivariable analysis for identifying predictors of wasting, child sex, ANC visit of the mother, diarrhea in the last 2 weeks, prelacteal feeding, and birth interval were found to be important predictors of wasting.

Male children were 1.89 times more at risk for wasting than female children (AOR = 1.89; CI = 1.01–3.54) and the risk of being wasted was 2.28 times higher for children who had experienced diarrhea in the last 2 weeks than in children who had not (AOR = 2.28; CI = 1.19–4.38). The result also revealed that children who were prelactated at birth were 5.28 times more likely to be wasted than children who did not experience prelacteal feeding at their birth (AOR = 5.28; CI = 2.45–11.36). The risk of being wasted for children whose mother received ANC service was 1.95 times higher than children whose mothers did not (AOR = 1.95; CI = 1.01–3.8). In addition, children who had <2 years' birth interval from the preceding child had 3.44 times higher risk to be wasted than children who had more than 3 years' birth interval (AOR = 3.44; CI = 1.38–8.58) (Table 11).

## 4. Discussion

### 4.1. Household Food Insecurity Status

Household Food Insecurity Access Scale (HFIAS) tool, which was developed by food and nutrition technical assistant (FANTA) project, was used to identify household food insecurity status of the area. In this study it was found that 75.8% of the households experienced some degree of food insecurity in the one month preceding the survey. This result was similar to the results of other studies conducted in Ethiopia, Wolayita and Oromia zones, where 74.2% and 73.1% of households face food insecurity, respectively [17, 23]. This may be due to the fact that these areas are found geographically adjacent to this study so that they have similar agroecology. Therefore, they are expected to have the same food production in amount and type.

This study also showed that 62.3% of the households reported that their household members have eaten smaller amount of food during the last four weeks and 66.1% missed the number of meals per day while 32.3 percent reported that they have no food of any kind to eat. This result was consistent with the study conducted in Sidama [24] that 67.4 percent have eaten smaller amount of food and 62.9 percent missed the number of meals per day while 29.8% experienced no food to eat.

Similarly the current study revealed that 10.7% of households reported sleeping without eating food. This finding was also in line with another study which is conducted in Ethiopia, Bangladesh, and Vietnam from the data obtained from the Alive & Thrive baseline survey. The result showed that 11.4% of households in Ethiopia faced sleeping without eating [25].

Results from Ethiopia showed that 16.9%, 34.1%, and 15.4% of the households were mild, moderate, and severely food insecure, respectively [25], and the overall house food insecurity was 66.4%. From the results of the present study it was found that 12.3%, 32.3%, and 31.0% of households were mild, moderate, and severely food insecure with overall food insecurity of 75.8%. The result of this study was slightly higher. The differences may be attributed to the fact that the above-mentioned study used a larger sample size and the samples were selected from different parts of the country having a varying geography, annual rain fall, and farm land size, so that they may have different amount and type of food production consequently; they will have a different extent of food insecurity. This implies that area specific surveys are better to understand the real situation of the area as the average result of different areas may not be the same with the actual figures of specific areas, since the problem of one area will possibly be hidden by another.

In contrary, in another study conducted in shone district [19], SNNPR revealed that 54.4 percent of households reported that they worried always about food shortage and 94.4% of respondents also cut the size of food and eat less than they felt, while 30.5% of adults were not eating the whole day. These findings are higher than the current study. This difference is probably attributed to the seasonal variation, as the two studies were conducted in different seasons and year and this implies that the prevalence HHFI may vary across seasons and years.

### 4.2. Nutritional Status of Children

To measure nutritional status of children, the height and weight of children were converted to *Z*-scores then the values were converted to three indices of nutritional status: underweight, stunting, and wasting according to WHO classification.

The result for nutritional status of children was 26.3% for underweight, 45.6% for stunting, and 14.6% for wasting. The regional prevalence of underweight, stunting, and wasting was 28.3%, 44.1%, and 7.6%, respectively, based on EDHS 2011 [16].

The result of the present study showed that the figures are comparable with the regional figure, EDHS. However, the figures, especially of wasting prevalence (14.6%), are slightly high in the current study. Similarly, severe stunting and wasting were very high as compared to the regional 22.9% and 2.8%. But severe underweight (10.5%) was alike with regional figure (9.6%). The high prevalence of malnutrition, mainly of wasting, in this study may be due to the fact most of the households in the study area face shortage of food during Belg season (February–May). The data was collected in this season. During this season, household's food reserve from the previous year gets smaller and the market price of the crops will increase. In addition, livestock body conditions and productivity will also be deteriorated. Furthermore, high proportion of child diarrhea in this study may possibly be attributed to the high prevalence of malnutrition.

Though the prevalence of underweight was similar with the national figure, it was very low compared to underweight prevalence reported by other studies conducted in Oromia (30.9%) [23], Tigray (38.3%) [21], west Gojam (49.2%) [26], Somale (47.7%) [20], and Jimma (34.2) [27]. However, the prevalence of stunting (45.6) was similar to the study of Oromia (47.6%) and west Gojam (43.2%), whereas it was higher than that of Jimma (40.4%) and Somali (34.4%) and the prevalence of wasting (14.8%) was similar to the above studies except for Jimma (5.1%) which was lower than the current study. The data for this research was collected in February, and mostly food insecurity in SNNPR areas is likely to be higher from January to March [28], so that there will be increase in acute child malnutrition. For most of the above literatures their study period is different from the current study and this may possibly be the reason for the difference in proportions.

### 4.3. Household Food Insecurity and Nutritional Status of Children

In this study the main purpose was to see the association of household food insecurity and nutritional status of children.

Regarding distribution of malnourished children between food secure and insecure households, the majority of malnourished children are found in food insecure households, though the prevalence of wasting was almost similar in food secure and insecure households which is comparable with the findings in Nepal [29].

The study of Nepal revealed that the prevalence rates of stunting and underweight were slightly higher among children from food insecure than among children from food secure households. In the present study the mean difference for HAZ and WAZ between secure and insecure households was significant (*P* < 0.05). However, the mean difference WHZ was not significant between the two groups. This result was also consistent with the finding of Nepal [29]. This similarity may be due to the fact that study of Nepal used the same tool as the current study to assess the food insecurity status of households and their prevalence of overall food insecurity 69.2% and their prevalence of malnutrition was slightly closer to findings of the current study (75.6%). Consequently the distribution of malnourished children in the two groups of households showed similarity with current study.

Unlike the present study, another study which was conducted in Brazil [30] showed that WHZ mean value was lower for children who lived in moderate to severe food insecurity compared to children in food secure households. This difference may be due to the fact that the method they used to assess household food insecurity status differs from the current study; as a result their prevalence of HHFI was also very low (48.6%) compared to the current study (75.8%).

In this study children living in food insecure household had 3.8 times higher risk of being underweight. It is expected that children in food insecure households may face reduced dietary variety or nutrient intake of food which will later result in malnutrition. Similarly the risk of being stunted, for the children living in food insecure household, was 6.7 times higher than those children living in food secure households. It is known that stunting is result of long time effect of under nutrition. Thus, children living in households, where food insecurity remains the same over the years, will face this problem and are expected to be stunted. However the present study assesses the situation of only one month preceding the survey and this was found to be the limitation of this study. Still this result was consistent with the study which was conducted in Ghana [22]; households that were food insecure were more likely than the households that were food secure to have children with chronic malnutrition. This can be explained as though HHFI varies across time, sometimes it may persist for a long time causing chronic malnutrition.

The current study showed that children living in food insecure households had no difference in wasting from children living in secure households. However, other literatures [18] have identified HHFI to be associated with acute malnutrition; their method to assess HHFI was completely different from the current study and as a result they yield a lower proportion of HHFI; this might be the reason for this disparity.

### 4.4. Other Factors Associated with Nutritional Status of Children

The fourth objective of our study was to see other factors associated with nutritional status of children other than HHFI, since child malnutrition is not the mere effect of HHFI.

In this study children who had diarrhea in the past 2 weeks preceding the survey were 2.5 times more at risk for underweight and 2.28 times more at risk for wasting than children who had not. This result was expected since diarrhea leads to malnutrition and malnutrition predisposes to diarrhea. It is also known that malnourished children will have frequent diarrheal episodes and lose weight and can quickly become malnourished [16]. This result was consistent with finding of other studies [16, 18].

Health status during pregnancy was also a risk factor for underweight that children whose mothers had no good health status during pregnancy were 2.23 times more likely to be underweight than children of mothers with good health status during pregnancy. Illness during pregnancy will prevent the mother from gaining weight; thus she will probably give a low birth weight baby and babies reported as very small or small at birth are much more likely to be underweight later in life [16].

Similarly children whose mothers did not receive ANC service were 2.8 times more at risk for underweight and 1.95 times more at risk for wasting than children whose mother did. This finding may be explained by high contact of mothers with the health service and mothers who attended ANC visit will find advice about good child feeding practice. Such mothers also have better heath seeking behavior and they are likely to take appropriate actions to improve the health status of their children, which is also important component of child nutrition. This result was in line with other study [31] conducted in Ethiopia.

According to WHO recommendation a child should start complementary feeding in 6–8 months of age. Study conducted in west Gojam [26] revealed that children who started complementary feeding lately were at risk for stunting. Similarly the result of this study indicated that children who had initiated complementary feeding early (before 6 months) were 2.41 times more at risk to be stunted than children who had started complementary feeding appropriately (6–8 months).

The national EDHS 2011 indicated that the prevalence of stunting increases as the age of child increases. Similarly the result of this study showed that the prevalence of stunting in children increased from 6–11 months to 36–47 months and the highest risk of stunting was seen among children aged 12–23 and 36–47 months but the risk was significant in age group of 36–47 months. Children aged 36–47 months were 2.17 times more likely affected by stunting as compared to children aged 6–11 months. This may be explained by the fact that foods for weaning are typically introduced to children in the older age group; thus it increases their exposure to infections and susceptibility to illness. This tendency, coupled with inappropriate or inadequate feeding practices, may contribute to faltering nutritional status among children in these age groups [16]. This result was consistent with other findings of Somale and Vietnam [18, 20].

In addition, the present study also indicated that children who were prelactated at birth were 5.28 times more likely to be wasted than children who were not. The practice of giving prelacteal feeds is discouraged because it limits the infant's frequency of suckling and exposes the baby to the risk of infection [16]. According to EDHS 2011 national report, nearly three children in every ten (27 percent) are given prelacteal feeds within the first three days of life. This finding agrees with previous literatures, which have shown that children who received prelacteal were more likely to be wasted [20].

A number of studies in Ethiopia and other developing countries suggest that malnutrition among boys is consistently higher than malnutrition among girls [18, 20, 30, 32]. Similar result was found in the present study; the odds of being wasted was 1.89 times higher among male children than among female children. This result could be due to unmeasured factors like parental gender preference or sex difference in feeding practice which needs further investigation.

Children who had <2 years' birth interval from the preceding child had 3.44 times higher risk to be wasted than children who had more than 3 years' birth interval. This result is consistent with others studies [16, 33]. Children born too soon after a previous birth, especially if the interval between the births is less than two years, are at increased risk for health problems. This may be due to the fact that child nutritional status is expected to improve with higher birth spacing as the mother would get enough time for care and feeding. When pregnancies are closely spaced, it is often the case that the mother will have little time to regain lost fat and nutrient stores. This implies that longer birth intervals improve the health status of both mother and child.

In several studies mother's education level had been associated with nutritional status of children [20, 32, 34]. We found that the prevalence of malnutrition decreased with increasing of educational status. However it was not statistically significant in adjusted analysis. This may be due to the fact that this study is conducted in rural setting; thus the educational status for most of the mothers is expected to be homogeneous, so that nutritional status of children may not show a difference in educational status.

Also size at birth of the child was significant in bivariate analysis with underweight. However the association disappeared when adjusting for other covariates. This could be due to the fact that data on birth size are collected by mother's perception towards their child's size during birth. Therefore there may be a recall bias.

Similarly colostrum feeding did not show association in this study unlike other studies [32]. It can be well explained by the fact that the child who is fed colostrum has got a natural immunity for childhood infections; however as the child age increases there may be other factors like diarrhea, inappropriate feeding practices, and so on which lead to malnutrition, so that the positive effect of colostrum feeding may possibly be hidden by these factors.

Different from other studies, no association was found between underweight, stunting, and wasting and the mother's age at birth in this study. As most of the mothers are not educated, they may not know their exact age, suggesting that the data on mother's age may probably be subjected to recall bias.

In the same way, all environmental health related characteristics investigated also failed to have a statistically significant association with any of the child nutritional status characteristics (stunting, wasting, and underweight). This is inconsistent with the reports of EDHS 2011 and other studies too [31, 33]. This may be related to the fact that child nutritional status is not susceptible to environmental health conditions since most of the households had latrine and use water from protected source.

### 4.5. Limitation of the Study

As the design of the study was cross-sectional, its ability to draw cause-effect relationship was limited. The analysis was based on the data that was collected during one season of the year, but the magnitude of household food insecurity may vary across seasons, so that data which shows seasonal variations may be needed to fully understand household food insecurity and its association with nutritional status (especially for stunting) of children.

Recall bias was also possible. Hence, some of the variables were based on a recall to a situations happening few weeks or months back from the actual data collection time. And this might explain high or low prevalence of household food insecurity. Despite these limitations, the results of this study will provide important contributions to the limited data available on household food insecurity and nutritional status of children in the study area.

## 5. Conclusion

Household food insecurity and the prevalence rates of stunting, underweight, and wasting, among children 6 to 59 months of age, were high in East Badawacho District, Hadiya Zone, and SNNPR. Though the prevalence of malnutrition in the area is similar to the regional and national figure, the finding of this study indicates that malnutrition is still an important major public health problem among children in the area.

Household food insecurity was significantly associated with underweight and stunting but not with wasting.

In addition, diarrhea, health status during pregnancy, and ANC visit come out to be significant risk factors for underweight. Initiation of complementary feeding and child age were found to be associated with stunting. Also child sex, diarrhea, prelacteal feeding, ANC visit, and birth interval were found to be risk factors for wasting. Thus, improving only household food security may be necessary but not sufficient to improve the nutritional status of children 6–59 months of age.

Programs to improve HFI along with other nutrition interventions may enable greater synergy and sustainable impacts in addressing childhood undernutrition than just nutrition-specific interventions.

## Figures and Tables

**Table 1:** Demographic and socioeconomic characteristics of parents at East Badawacho, South Ethiopia, February 2014.

Variable	Category	Frequency	Percent
Head of the household	Female	49	9.9
	Male	447	90.1
	Total	496	100.0

Mother age	<24	50	10.1
	25–34	355	71.6
	>35	91	18.3
	Total	496	100.0

Educational level of mother	No education	217	43.8
	Primary	203	40.9
	Secondary and above	76	15.3
	Total	496	100.0

Educational level of father	No education	123	24.8
	Primary	244	49.3
	Secondary and above	128	25.9
	Total	495	100.0

Occupation of mother	Housewife	411	82.9
	Farmer	14	2.8
	Merchant	33	6.7
	Private org employee	4	0.8
	Government employee	22	4.4
	Daily laborer	12	2.4
	Total	496	100.0

Occupation of father	Farmer	263	58.8
	Daily worker	50	11.2
	Merchant	17	3.8
	Private org employee	41	9.2
	Government employee	76	17.0
	Total	447	100.0

Ethnicity	Hadiya	348	70.2
	Kembata	53	10.7
	Wolayita	53	10.7
	Halaba	35	7.1
	Other^*∗*^	7	1.4
	Total	496	100

Religion	Protestant	367	74.0
	Orthodox	59	11.9
	Muslim	39	7.9
	Catholic	31	6.3
	Total	496	100.0

Number of under-five children	1 child	234	47.2
	2 children	214	43.1
	>3 children	48	9.7
	Total	496	100.0

Family size	<5	147	29.6
	>6	349	70.4
	Total	496	100

Wealth index	Very poor	98	20.0
	Poor	100	20.4
	Middle	96	19.6
	Rich	99	20.2
	Very rich	98	20.0

^*∗*^Other = Amara (3), Oromo (2), and Tigre (2).

**Table 2:** Obstetric characteristics of the mothers in East Badawacho, South Ethiopia, February 2014.

Variable	Category	Frequency	Percent
Age at marriage	<19	227	45.8
	>20	269	54.2
	Total	496	100.0

Children ever born	≤4	298	60.1
	>5	198	39.9
	Total	496	100.0

Marital status	Single	2	0.4
	Married	446	89.9
	Divorced	25	5.0
	Widowed	23	4.6
	Total	496	100.0

Extra food during pregnancy	Yes	150	30.2
	No	346	69.8
	Total	496	100.0

Health status during the pregnancy	Good	410	82.7
	Sick	86	17.3
	Total	496	100.0

ANC visit	Yes	349	70.4
	No	147	29.6
	Total	496	100.0

**Table 3:** Demographic and health related characteristics of children aged 6–59 months in East Badawacho District, South Ethiopia, February 2014.

Variables	category	Frequency	Percent
Child's sex	Male	245	49.4
	Female	251	50.6
	Total	496	100.0

Child age	6–11	67	13.5
	12–23	131	26.4
	24–35	142	28.6
	36–47	107	21.6
	48–59	49	9.9
	Total	496	100

Birth interval	1 year	76	17.0
	2 years	221	49.4
	>3 years	150	33.6
	Total	447	100.0

Birth order	1 year	49	9.9
	2-3 years	150	30.2
	4-5 years	179	36.1
	6+ years	118	23.8
	Total	496	100.0

Place of delivery	Home	413	83.3
	Health institution	83	16.3
	Total	496	100.0

Delivery attendance	TBA	372	75.0
	Health personnel	84	16.9
	Family	40	8.1
	Total	496	100.0

Diarrhea	Yes	165	33.3
	No	331	66.7
	Total	496	100.0

Fever	Yes	171	34.5
	No	325	65.5
	Total	496	100.0

Other serious illness	Yes	66	13.3
	No	430	86.7
	Total	496	100

Birth weight/size at birth	Small	85	17.1
	Average	234	47.2
	Larger than average	177	35.7
	Total	496	100.0

Respiratory disease	Yes	82	16.5
	No	414	83.5
	Total	496	100.0

Measles	Yes	21	4.2
	No	475	95.8
	Total	496	100

**Table 4:** Child caring practice in East Badawacho District, Hadiya zone, South Ethiopia, February 2014.

Variable	Category	Frequency	Percent
Breastfeed	Yes	419	84.5
	No	77	15.5
	Total	496	100.0

Initiation of BF	Immediately	218	52.0
	Hours	166	39.6
	Days	35	8.4
	Total	419	100.0

Feed colostrum	Yes	270	64.4
	No	149	35.6
	Total	419	100.0

Continuation of BF	12–23 months	187	44.6
	<11 months	117	27.9
	>24 months	115	27.4
	Total	419	100.0

Prelacteal feeding	No	415	83.7
	Yes	81	16.3
	Total	496	100.0

Initiation of CF	6–8 months	367	74.0
	<5 months	100	20.2
	>9 months	29	5.8
	Total	496	100.0

Frequency of feeding	<3 times	323	65.1
	≥4 times	173	34.9
	Total	496	100

Wash your hands feeding the child	Yes	472	95.2
	No	24	4.8
	Total	496	100

Bath taking of the child	Daily	348	70.2
	Weekly	148	29.8
	Total	496	100

Taking care of the baby feeding	Mother	450	90.7
	Sister	24	4.8
	Grand mother	13	2.6
	Housemaid	9	1.8
	Total	496	100

Immunization status	Yes	453	91.3
	No	43	8.7
	Total	496	100.0

Vitamin A	Yes	441	88.9
	No	55	11.1
	Total	496	100

Deworming	Yes	286	57.7
	No	210	42.3
	Total	496	100.0

**Table 5:** Prevalence of household food insecurity in East Badawacho District, South Ethiopia, February 2014.

Variable	Category	Frequency	Percent
Worry about food	No	179	36.1
	Rarely	129	26.0
	Sometimes	148	29.8
	Often	40	8.1

Unable to eat preferred foods	No	168	33.9
	Rarely	149	30.0
	Sometimes	139	28.0
	Often	40	8.1

Eat just a few kinds of foods	No	166	33.5
	Rarely	151	30.4
	Sometimes	131	26.4
	Often	48	9.7

Eat foods they really do not want to eat	No	204	41.1
	Rarely	157	31.7
	Sometimes	116	23.4
	Often	19	3.8

Eat a smaller meal	No	187	37.7
	Rarely	165	33.3
	Sometimes	118	23.8
	Often	26	5.2

Eat fewer meals in a day	No	165	33.3
	Rarely	176	35.5
	Sometimes	133	26.8
	Often	22	4.4

No food of any kind in the household	No	336	67.7
	Rarely	74	14.9
	Sometimes	70	14.1
	Often	16	3.2

Go to sleep hungry	No	443	89.3
	Rarely	28	5.6
	Sometimes	23	4.6
	Often	2	0.4

Go a whole day and night without eating	No	467	94.2
	Rarely	15	3.0
	Sometimes	13	2.6
	Often	1	0.2

Food aid	Yes	46	9.3
	No	450	90.7

**Table 6:** Nutritional status of children in East Badawacho District, South Ethiopia, February 2014.

Nutritional status of children	Number	Percent
Underweight (*n* = 485)		
Normal	358	73.8
Moderate	76	15.7
Severe	51	10.5

Stunting (*n* = 496)		
Normal	270	54.4
Moderate	93	18.8
Severe	133	26.8

Wasting (*n* = 485)		
Normal	414	85.4
Moderate	32	6.6
Severe	39	8.0
O-edema (*n* = 496)	11	2.2

**Table 7:** Stunting, wasting, and overweight among children aged 6–59 months from food secure and food insecure households, East Badawacho District, South Ethiopia, February 2014.

Nutritional status of children		All children		Household food insecurity status			
				HHF secure		HHF insecure	
		*N*	%	*N*	%	*N*	%
WAZ	Normal	358	73.8	107	89.2	251	68.8
	Moderate	76	15.7	8	6.7	68	18.6
	Severe	51	10.5	5	4.2	46	12.6
	Total	485	100	120	100	365	100
	*Mean (±SD)* ^†^	*−1.13 (±1.46)*		*−0.70 (±1.27)*		*−1.28 (±1.49)* ^*∗∗*^	

HAZ	Normal	270	54.4	99	82.5	171	45.5
	Moderate	93	18.8	13	10.8	80	21.3
	Severe	133	26.8	8	6.7	125	33.2
	Total	496	100	120	100	376	100
	*Mean (±SD)* ^†^	*−1.61 (±2.29)*		*−0.78 (±1.93)*		*−1.88 (±2.34)* ^*∗∗*^	

WHZ	Normal	414	85.4	103	85.8	311	85.2
	Moderate	32	6.6	4	3.3	28	7.7
	Severe	39	8.0	13	10.8	26	7.1
	Total	485	100	120	100	365	100
	*Mean (±SD)* ^†^	*−0.12 (±1.88)*		*−0.03 (±1.99)*		*−0.15 (±1.84)*	

^*∗∗*^ *P* < 0.05, for the difference between food insecure and food secure households, independent sample *t*-test.

^†^Mean and SD of *Z*-score.

**Table 8:** Association of HHFI with nutritional status of children measured by stunting, underweight, and wasting in East Badawacho District, South Ethiopia, February 2014.

Nutritional status of children	HHFI status	Yes	No	COR (CI)	*P* value	AOR (CI)^*∗*^
		*N* (%)	*N* (%)			
Underweight	Food insecure	114 (89.8)	251 (70.1)	3.74 (2.02–6.93)^*∗∗*^	0.001	3.82 (1.78–8.19)^*∗∗*^
	Food secure	13 (10.2)	107 (29.9)	1		1

Stunting	Food insecure	205 (90.7)	171 (63.3)	5.65 (3.38–9.44)^*∗∗*^	<0.001	6.70 (3.71–12.11)^*∗∗*^
	Food secure	21 (9.3)	99 (36.7)	1		1

Wasting^†^	Food insecure	54 (76.1)	311 (75.1)	1.05 (0.58–1.89)	0.84	
	Food secure	17 (23.9)	103 (24.9)	1		

^*∗*^Adjusted for other independent variables (Tables 9, 10, and 11), ^*∗∗*^*P* < 0.05.

^†^HHFI was not included in multivariable analysis since *P* value was above 0.2 in bivariate analysis with wasting.

**Table 9:** Factors associated with nutritional status of children, 6–59 months of age, as measured by underweight in East Badawacho District, South Ethiopia, February 2014.

Variable	Underweight status		COR (95.0% CI)	AOR (95.0% CI)
	Underweight	Normal		
	*N* (%)	*N* (%)		
*HHFI status*				
Food insecure	114 (89.8)	251 (70.1)	3.74 (2.02–6.93)^*∗∗*^	3.82 (1.78–8.19)^*∗∗*^
Food secure	13 (10.2)	107 (29.9)	1	1

*Diarrhea*				
Yes	60 (47.2)	97 (27.1)	2.41 (1.58–3.66)^*∗∗*^	2.51 (1.52–4.13)^*∗∗*^
No	67 (52.8)	261 (72.9)	1	1

*Size at birth*				
Larger than average	45 (35.4)	129 (36.0)	1	1
Average	56 (44.1)	173 (48.3)	0.93 (0.59–1.46)	0.76 (0.43–1.32)
Small	26 (20.5)	56 (15.6)	1.33 (0.75–2.36)	0.76 (0.37–1.55)

*Prelacteal feeding*				
Not prelactated	96 (75.6)	312 (87.2)	1	1
Prelactated	31 (24.4)	46 (12.8)	2.19 (1.31–3.64)^*∗∗*^	1.81 (0.97–3.33)

*Health status during pregnancy*				
Sick	37 (29.1)	49 (13.7)	2.59 (1.59–4.22)^*∗∗*^	2.23 (1.27–3.94)^*∗∗*^
Good	90 (70.9)	309 (86.3)	1	1

*Extra food during pregnancy*				
No	98 (22.8)	240 (33.0)	1.66 (1.03–2.65)^*∗∗*^	0.98 (0.55–1.74)
Yes	29 (77.2)	118 (67.0)	1	1

*Birth interval*				
<2 years (<23 m)	26 (22.8)	48 (14.8)	1.80 (0.97–3.34)	1.41 (0.7–2.85)
2 years (24–35 m)	55 (48.2)	166 (51.2)	1.10 (0.67–1.81)	1.08 (0.62–1.87)
>3 years (>36 m)	33 (28.9)	110 (34.0)	1	1

*Mother's age*				
<24	19 (15.0)	30 (8.4)	1	1
25–34	88 (69.3)	260 (72.6)	0.53 (0.28–0.99)^*∗∗*^	0.48 (0.19–1.19)
>34	20 (15.7)	68 (19.0)	0.46 (0.21–0.99)^*∗∗*^	0.43 (0.15–1.24)

*ANC visit*				
No	56 (44.1)	87 (24.3)	2.45 (1.60–3.76)^*∗∗*^	2.8 (1.66–4.7)^*∗∗*^
Yes	71 (55.9)	271 (75.7)	1	1

^**∗∗**^ *P* value < 0.05.

**Table 10:** Factors associated with nutritional status of children, 6–59 months of age, as measured by stunting (height for age) in East Badawacho Woreda, South Ethiopia, February 2014.

Variable	Stunting status		COR (95.0% CI)	AOR (95.0% CI)
	Stunted	Normal		
	*N* (%)	*N* (%)		
*HHFI status*				
Food insecure	205 (90.7)	171 (63.3)	5.65 (3.38–9.44)	6.70 (3.71–12.11)^*∗∗*^
Food secure	21 (9.3)	99 (36.7)	1.00	1.00

*Child sex*				
Male	112 (49.6)	133 (49.3)	1.01 (0.71–1.44)	0.88 (0.61–1.31)
Female	114 (50.4)	137 (50.7)	1.00	1.00

*Child age in months*				
6–11	32 (14.2)	35 (13.0)	1.00	1.00
12–23	60 (26.5)	71 (26.3)	0.92 (0.51–1.66)	1.14 (0.61–2.14)
24–35	52 (23.0)	90 (33.3)	0.63 (0.35–1.14)	0.84 (0.44–1.59)
36–47	64 (28.3)	43 (15.9)	1.63 (0.88–3.01)	2.17 (1.11–4.28)^*∗∗*^
48–59	18 (8.0)	31 (11.5)	0.63 (0.29–1.35)	0.93 (0.41–2.08)

*Initiation of CF*				
<5 months	57 (25.2)	43 (15.9)	1.75 (1.12–2.74)^*∗∗*^	2.41 (1.36–4.27)^*∗∗*^
6–8 months	158 (69.9)	209 (77.4)	1.00	1.00
>9 months	11 (4.9)	18 (6.7)	0.81 (0.37–1.76)	1.05 (0.43–2.55)

*Education of mother*				
No education	110 (48.7)	107 (39.6)	2.09 (1.21–3.61)^*∗∗*^	0.95 (0.48–1.86)
Primary	91 (40.3)	112 (41.5)	1.65 (0.95–2.88)	0.76 (0.38–1.52)
Secondary and above	25 (11.1)	51 (18.9)	1.00	1.00

*Ever breastfeed*				
No	35 (15.5)	42 (15.6)	0.99 (0.61–1.62)	0.77 (0.39–1.54)
Yes	191 (84.5)	228 (84.4)	1.00	1.00

*Frequency of feeding*				
<3 times	159 (70.4)	164 (60.7)	1.53 (1.05–2.23)^*∗∗*^	0.99 (0.62–1.56)
≥4 times	67 (29.6)	106 (39.3)	1.00	1.00

^*∗∗*^ *P* value < 0.05.

**Table 11:** Factors associated with nutritional status of children, 6–59 months of age, as measured by wasting (height for age) in East Badawacho District, South Ethiopia, February 2014.

Variable	Wasting status		COR (95.0% CI)	AOR (95.0% CI)
	Wasting	Normal		
	*N* (%)	*N* (%)		
*Child sex*				
Male	40 (56.3)	200 (48.3)	1.38 (0.83–2.29)	1.89 (1.01–3.54)^*∗∗*^
Female	31 (43.7)	214 (51.7)	1	1

*Diarrhea*				
Yes	34 (47.9)	123 (29.7)	1	1
No	37 (52.1)	291 (70.3)	2.17 (1.30–3.62)^*∗∗*^	2.28 (1.19–4.38)^*∗∗*^

*Prelacteal feeding*				
Not prelactated	48 (67.6)	360 (87.0)	1	1
Prelactated	23 (32.4)	54 (13.0)	3.19 (1.8–5.67)	5.28 (2.45–11.36)^*∗∗*^

*Continuation of BF*				
<11 months	15 (23.1)	98 (28.5)	0.59 (0.3–1.13)	0.66 (0.31–1.40)
12–23 months	38 (58.5)	147 (42.7)	1	1
>24 months	12 (18.50)	99 (28.8)	0.47 (0.23–0.94)^*∗∗*^	0.48 (0.21–1.07)

*Colostrum feeding*				
No	30 (46.2)	115 (33.4)	1.70 (0.99–2.92)	1.33 (0.69–2.57)
Yes	35 (53.8)	229 (66.6)	1	1

*ANC visit*				
No	34 (47.9)	109 (26.3)	2.57 (1.54–4.30)^*∗∗*^	1.95 (1.01–3.80)^*∗∗*^
Yes	37 (52.1)	305 (73.7)	1	1

*Birth interval*				
<2 years (<23 m)	19 (27.9)	55 (14.9)	3.45 (1.6–7.5)	3.44 (1.38–8.58)^*∗∗*^
2 years (24–35 m)	36 (52.9)	185 (50.0)	1.94 (0.99–3.81)	2.16 (0.97–4.80)
>3 years (>36 m)	13 (19.1)	130 (35.1)	1	1

*Frequency of feeding*				
<3 times	53 (74.6)	262 (63.3)	1.71 (0.96–3.02)	1.14 (0.54–2.39)
≥4 times	18 (25.4)	152 (36.7)	1	1

^*∗∗*^ *P* value < 0.05.

## Abbreviations

FANTA: Food and nutrition technical assistant
HAZ: Height for age *Z*-score
HFIAS: Household Food Insecurity Access Scale
HHFI: Household food insecurity
WAZ: Weight for age *Z*-score
WHZ: Weight for height *Z*-score.
